# Recent advances in targeting protein kinases and pseudokinases in cancer biology

**DOI:** 10.3389/fcell.2022.942500

**Published:** 2022-07-22

**Authors:** Kristina Riegel, Parthiban Vijayarangakannan, Petros Kechagioglou, Katarzyna Bogucka, Krishnaraj Rajalingam

**Affiliations:** ^1^ Cell Biology Unit, University Medical Center Mainz, JGU-Mainz, Mainz, Germany; ^2^ Svastia Genetics, Future Business Centre, Cambridge, United Kingdom

**Keywords:** kinome, RAF, MAPK, CDK4/6, inhibitors, cancer, molecular switch, allosteric regulation

## Abstract

Kinases still remain the most favorable members of the druggable genome, and there are an increasing number of kinase inhibitors approved by the FDA to treat a variety of cancers. Here, we summarize recent developments in targeting kinases and pseudokinases with some examples. Targeting the cell cycle machinery garnered significant clinical success, however, a large section of the kinome remains understudied. We also review recent developments in the understanding of pseudokinases and discuss approaches on how to effectively target in cancer.

## Introduction

Kinases are a key class of enzymes, which catalyze the covalent attachment of the gamma-phosphate of ATP to their various targets including proteins, lipids, and nucleotides. Protein kinases represent one of the largest gene families in eukaryotes with more than 518 protein kinases forming the human kinome (Manning et al., 2002). Among them, protein kinases phosphorylating either tyrosine (tyrosine-specific protein kinases) or serine/threonine (Ser-/Thr-specific protein kinases) are the predominant ones (Martin et al., 2010). Phosphorylation of a protein can affect its function in a variety of ways, such as enhancing or inhibiting its biological activity in terms of enzymatic reactions, transcription, or translation, or affecting the stability, complex formation, or cellular localization of a protein (Roskoski, 2015). Thus, protein phosphorylation is a powerful tool for regulating almost all cellular functions. To name just a few examples, protein kinases play a central role in controlling cell division, cell movement, cell death, transcription, and cell metabolism (Manning et al., 2002; Roskoski, 2015). Further, dysregulation or mutation of protein kinases are linked to many human diseases. The extent of the correlation between kinase dysfunction and diseases was underlined, by the study of Manning et al. (2002), in which they were mapping all kinase genes to chromosomal loci revealing that 164 kinases map to amplicons seen in tumors and 80 kinases map to amplicons associated with other major diseases (Manning et al., 2002). Melnikova and Golden (2004) even suggest that protein kinases are directly or indirectly linked to 400 human diseases making them to attractive targets for therapeutic strategies (Melnikova and Golden, 2004). It took until 2001, after the first description of enzymatic phosphorylation in 1954 by Burnett and Kennedy (1954); Roskoski (2015) and the identification of Rous sarcoma virus (v-Src) as the first transforming factor in 1978 (Collett and Erikson, 1978), for imatinib to be approved as the first small molecule kinase inhibitor by the FDA, which in turn opened the way for further targeted therapeutics in clinical oncology (Cohen, 2002; Roskoski, 2015; Wu et al., 2016; Cohen et al., 2021). By 2021, the number of FDA-approved kinase inhibitors against neoplasm has increased to a total of 58 (Roskoski, 2022), with the majority targeting receptor protein-tyrosine kinases. Newer inhibitors approved by the FDA in 2021 include the MET kinase inhibitor tepotinib (Mathieu et al., 2022), the VEGFR kinase inhibitor tivozanib (Chang et al., 2022), and the EGFR kinase inhibitor mobocertinib (Markham, 2021). Furthermore, the inhibitor trilaciclib was approved to reduce chemotherapy-induced myelosuppression. Trilaciclib targets the serine/threonine kinases CDK4/6 (Powell and Prasad, 2021), which we will discuss in more detail later. Also worth mentioning are the latest developments in BTK inhibitors. All three FDA-approved BTK inhibitors are covalent inhibitors that bind to the nucleophilic Cys481 and are used to treat B-cell-associated malignancies (Guo et al., 2019; Gabizon and London, 2020). However, because there is a need for more selective BTK inhibitors, alternative binding modes of BTK have been exploited. This in turn has led to the development of CGI1746, which reversibly binds BTK in an inactive conformation (Di Paolo et al., 2011). Based on this compound, optimized inhibitors such as the reversible inhibitor fenebrutinib and the covalent inhibitor remibrutinib have been developed and are currently under clinical investigation (Angst et al., 2020; Gabizon and London, 2020).

## From structure to function to inhibitors

To develop effective targeting strategies and rational kinase inhibitors, it is important to know the basic structure of kinase domains and how structural features in turn affect function. A typical protein kinase domain comprises of a small, mostly β-stranded N-lobe and a larger, α-helical C-lobe, which are connected by a short hinge region. As reviewed by Fabbro et al. (2015), the N-lobe harbors the glycine-rich loop (P-loop) that coordinates the phosphates of ATP and a single α-helix (C-helix) that can occupy different positions, contributing to the formation of an active or inactive kinase state (Fabbro et al., 2015). More precisely, the ‘α-helix-in’ position facilitates the interaction between the active site Lys and the Glu from the α-helix required for efficient catalysis (Cowan-Jacob, 2006; Fabbro et al., 2015). Deep in the ATP pocket is an additional residue, called “gatekeeper”, which restricts access to a pre-existing pocket and whose mutation leads to drug resistance (Fabbro et al., 2015). The C-lobe contains most of the catalytic machinery including the Y/HRD motif of the catalytic loop and the DFG motif. While the Y/HRD motif correctly positions the acceptor group to allow the contacts important for an efficient catalysis, the DFG motif brings the Asp in the correct position. In many instances, the DFG motif can take an “in” position where Phe rotates out of the ATP binding pocket and brings Asp to the site to coordinate Mg^2+^. In the DFG “out” position, however, Phe occupies the pocket (Fabbro et al., 2015). A generally accepted kinase activation model has been proposed in which kinases possess a series of highly conserved residues that form two parallel columns upon activation, termed regulatory and catalytic spines (Kornev et al., 2006).

As the example of RAF kinases shows, the activation of kinases can also be allosterically linked to the dimerization of the kinase. Here, the leucine residue of the α-helix involved in the regulatory spine formation is adjacent to an arginine residue, that is, part of the conserved RKTR motif at the dimer interface and thus plays a role in RAF dimer formation (Durrant and Morrison, 2018). Consequently, RAF dimerization pushes the α-helix to the “in” position, which contributes to the alignment of the regulatory spine and to the global stabilization of the active kinase conformation (Durrant and Morrison, 2018).

Protein kinase inhibitors have been classified as type I, II, and III inhibitors. Type I inhibitors bind reversibly at the ATP-binding pocket and extend into proximal regions to achieve greater selectivity. Another common feature is that these inhibitors bind the kinase in a DFG “in,” active state. A typical example of a type I inhibitor is the EGFR inhibitor gefinitib (Roskoski, 2016). In addition to type I inhibitors, there are type 1.5 inhibitors such as the RAF inhibitor vemurafenib, which are ATP-competitive inhibitors, that bind the kinase with a DFG “in” but α-helix “out” conformation (Tsai et al., 2008; Fabbro et al., 2015). In contrast to type I inhibitors, type II inhibitors bind to the DFG “out” conformation. Here, the ATP binding site as well as an additional hydrophobic pocket adjacent to the ATP pocket, which is present in the DFG “out” conformation, are preferentially occupied by type II inhibitors (Fabbro et al., 2015). An example for a type II inhibitor is imatinib (Nagar et al., 2002), the first FDA-approved kinase inhibitor, which inhibits BCR-Abl tyrosine kinase and is used for the treatment of CML (Roskoski, 2015). Inhibitors that do not bind the ATP binding site but an allosteric site are called type III or allosteric inhibitors. The advantage of type III inhibitors is that they do not compete with ATP binding and that they show a higher degree of selectivity since binding sites and regulatory mechanisms are targeted, which are unique for the respective kinase (Roskoski, 2016). A typical example for a type III inhibitor is trametinib, a MEK inhibitor approved for the treatment of melanoma.

Targeting strategies can be adapted dependent on what kind of problems are occurring with the developed inhibitors. A good example is the development of RAF inhibitors. There are three RAF isoforms (ARAF, BRAF and CRAF), which are part of the classical MAPK (mitogen-activated protein kinase) pathway RAF-MEK1/2-ERK1/2. MAPKs are a class of ubiquitously expressed serine/threonine-specific protein kinases. The mammalian family of MAPKs is represented by 14 members and organized into seven signaling pathways with the archetypal, RAS-dependent RAF-MEK1/2-ERK1/2 module at the forefront of the drug development research in cancer (Cargnello and Roux, 2011; Degirmenci et al., 2020).

Upon growth factor stimulation, RAS is activating this pathway, which regulates fundamental cellular processes including migration, cell survival and cell division. The classical MAPK pathway gained enormous interest since this pathway is often deregulated in human cancer with RAS and BRAF being among the most frequently mutated oncogenes. Therefore, much time has been invested in the development of drugs targeting this signaling pathway. Although the initial success of the RAF inhibitor vemurafenib has been impressive, rapidly developing drug resistance and the occurrence of secondary malignancies were of concern. Further studies revealed that the first-generation RAF inhibitors such as vemurafenib induced paradoxical MAPK activation in a BRAF mutation-free context by triggering RAF dimerization. To overcome this effect, next generation inhibitors such as pan-RAF inhibitors have been developed, which bind RAF kinases in a DFG “out”/α-helix “in” state thus inhibiting monomeric and dimeric RAF with equal efficiency (Durrant and Morrison, 2018). Drugs targeting the RAF dimer interface such as the PLX8394 represent an alternative approach to prevent the drug-induced paradoxical MAPK activation (Yao et al., 2019). As we found in our own study, a change in the targeting strategy could lead to different side effects than first-generation inhibitors, such as impairment of the function of certain immune cells (Riegel et al., 2019), which needs to be carefully considered in further inhibitor development.

Instead of aiming at inhibition of the kinase activity, new therapeutics induce degradation of the target by recruiting an E3 ligase. These so-called PROteolysis TArgeting Chimeras (PROTACs) are heterobifunctional molecules that bring the target and the E3 ligase in proximity, causing ubiquitination and subsequent degradation by the ubiquitin-proteasome system (Khan S. et al., 2020). This strategy would also be useful to target the kinase-independent functions.

**FIGURE 1:**
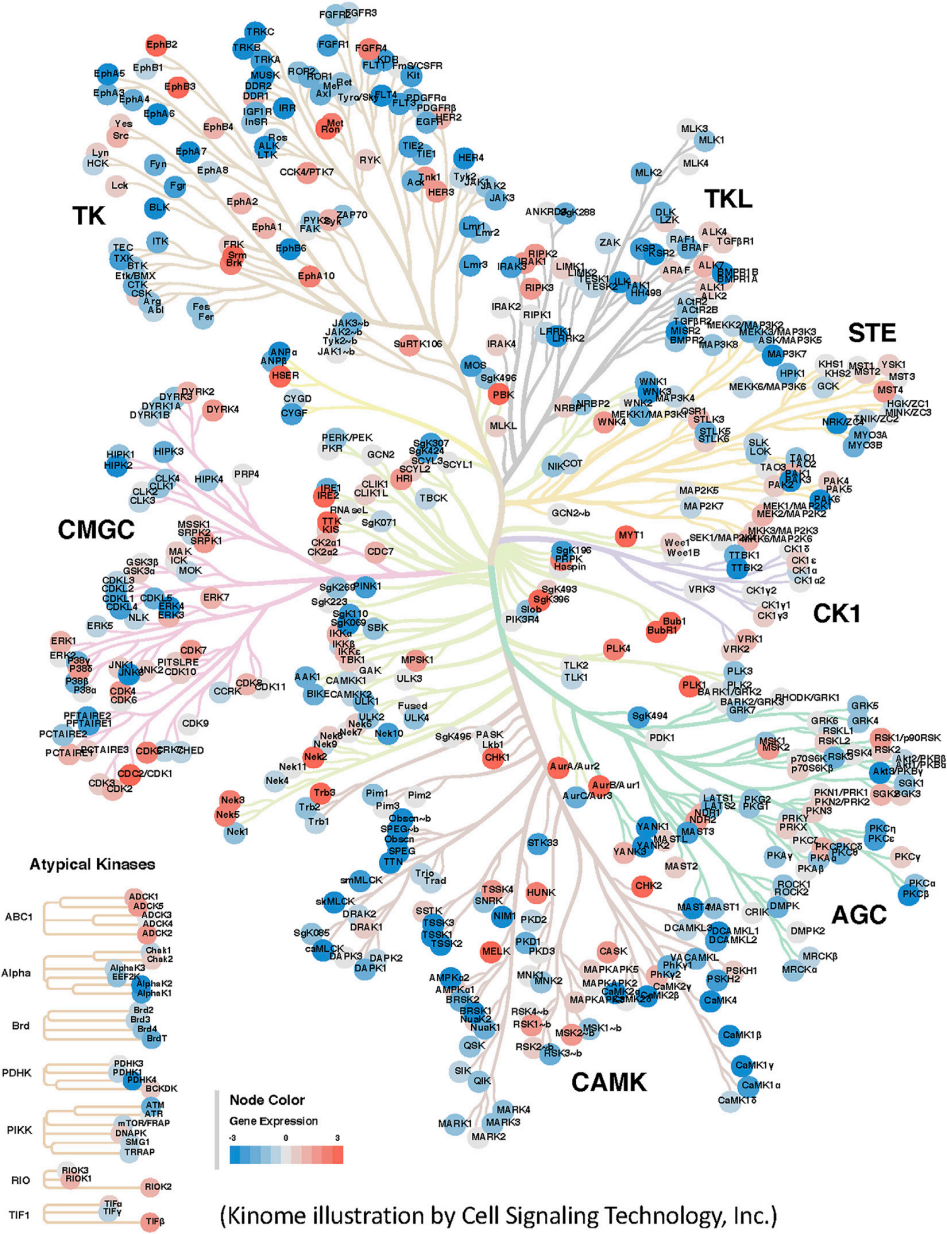
Visualization of differentially expressed kinases in cancer. Differential expression of kinases in Colon Adenocarcinoma was estimated using 100 TCGA-COAD RNA-Seq tumor samples (cases) and 165 normal samples (controls) derived from various human tissues by (Suntsova et al., 2019). Raw gene counts of both samples were downloaded from the Genomic Data Commons and Sequence Read Archive (SRA ID: SRP163252) respectively. DEseq2 R package was used to normalize the data and estimate the differential expression of genes. The log2 ratio of differentially expressed kinases were then extracted and used as quantitative input data within the CORAL web application (Metz et al., 2018). A few prominent differentially expressed kinases such as MET and RON as one of the highly expressed kinases in the results.

In addition to the development of novel drugs, researchers studying the kinome now increasingly rely on its bioinformatics visualization, which can be used in different ways. For example, pathways in which kinases are involved may be visualized using the Reactome database. Moreover, using Kinome trees researchers can analyze the range of highly expressed kinases in diseases such as cancer (Figure 1). For advanced research, tumor samples could be also classified according to molecular subtypes (e.g., TCGA-COAD subtypes) to identify biomarkers or kinase drug targets of interest against specific cancer types or subtypes (Guinney et al., 2015). While it is useful to identify a single kinase target, that is highly expressed in a number of molecular subtypes or cancers, it is possible that a particular kinase is only highly expressed in one of the molecular subtypes, so a corresponding drug target for that subtype may be promising.

## Targeting hallmarks of cancer using CDK4/6 inhibitors as an example

Since hyperactivation of cell cycle proteins and uncontrolled proliferation are hallmarks of cancer, one of the strategies is to target the cell cycle itself. The cyclin-dependent kinases (CDKs) are serine/threonine kinases that regulate cell cycle progression (Malumbres and Barbacid, 2001). CDK4/6 are crucial regulators of the G1/S transition, and their activity is activated by the D-type cyclins D1, D2, and D3 (cyclin D) (Morgan, 1997; Sherr and Roberts, 2004). For cells to enter S phase from G1 phase, CDK4 cyclin D1 and subsequently CDK2 cyclin E must hyperphosphorylate and thereby inactivate the retinoblastoma-associated protein (Rb) (Weinberg, 1995). Inactivation of pRb leads to the release of the transcription factor E2F from the Rb-E2F complex that actively represses transcription of cell cycle genes (Chellappan et al., 1991). Therefore, CDK4/6 enzymes are considered promising targets in cancer therapy (Hanahan and Weinberg, 2011; Otto and Sicinski, 2017), and the anti-tumor effect of small molecule CDK4/6 inhibitors is based on blocking the phosphorylation of the tumor suppressor retinoblastoma (Rb), and induction of G1/S arrest in tumor cells (Knudsen et al., 2019) (Luo et al., 1998).

Developing compounds with selectivity for CDK4/6 over other CDKs has been challenging, but Pyrido(2,3-d)pyrimidinone finally showed high selectivity for CDK4 compared to other CDKs (Barvian et al., 2000) and served as a precursor compound for the development of the FDA-approved ATP-competitive inhibitors palbociclib (Ibrance, PD0332991), ribociclib (Kisqali, LEE011), abemaciclib (Verzenio, LY2835219), and trilaciclib (G1 therapeutics, G1T28-1) (Beaver et al., 2015; Walker et al., 2016; Kim, 2017; Syed, 2017; Powell and Prasad, 2021). Palbociclib and ribociclib are both approved for the treatment of hormone receptor-positive (HR+), human epidermal growth factor receptor 2 (HER2)-negative advanced breast cancer patients in combination with endocrine therapy. Abemaciclib is the only one that can be used as monotherapy in adult patients with disease progression following endocrine therapy and prior chemotherapy in the advanced breast cancer, or in combination with fulvestrant in case of disease progression after endocrine therapy. CDK4/6 inhibitors have changed the treatment strategy for HR+/HER2− advanced breast cancer patients, and more than 17 CDK4/6 inhibitors in combination with various drugs, are now being tested or have been tested in clinical trials.

### Mechanism of action

Several studies have suggested that the CDK4/6 inhibitors bind to the ATP-binding pocket of CDK4 and CDK6 (Mariaule and Belmont, 2014) and block the cell proliferation in a wide range of tumors and reduce tumor growth in cancer xenograft models (Fry et al., 2004; Marzec et al., 2006; Logan et al., 2013; Tate et al., 2014; Gong et al., 2017; Kim et al., 2018). Another potential mechanism, after prolonged treatment with CDK4/6 inhibitors, may be the induction of RB1 and FOXM1-dependent senescence (Dean et al., 2010; Anders et al., 2011; Vijayaraghavan et al., 2017). Senescence has been demonstrated as a desired mechanism of cell growth inhibition by blocking the cell cycle progression (Braig et al., 2005). However, one of the chemotoxicity-induced mechanisms of chemotherapy and radiotherapy is the excessive induction of senescence in non-malignant cells (Baar et al., 2017; Murali et al., 2018; Yao et al., 2020). The main regulator of these detrimental properties is the transcription factor NF-κB, which triggers the “senescence-associated secretory phenotype” in conventional cancer treatments (Rodier et al., 2009; Chien et al., 2011). Meanwhile, a new study has found that CDK4/6 inhibitors induced senescence in non-malignant cells without toxicity, which was dependent on the transcriptional activity of the tumor suppressor p53 rather than NF-κB and therefore lacked most of the common pro-inflammatory senescence-associated secretory phenotype factors responsible for several adverse reactions (Wang et al., 2022).

### Resistance and other limitations of CDK4/6 inhibitors

The discovery of CDK4/6 inhibitors is considered a game-changer in cancer treatment. However, intrinsic or acquired resistance of HR+/HER2- metastatic breast cancer to clinically approved CDK4/6 inhibitors are responsible for disease progression in the majority of patients. Identifying reliable prognostic biomarkers that enable novel treatment combinations is the key to treatment success (Shah et al., 2018). Multiple studies have demonstrated that one of the most common mechanisms of cancer cell resistance to CDK4/6 inhibitors is loss of function of Rb (mutation, hyperphosphorylation or deletion) (Dean et al., 2012; Malorni et al., 2016; Vijayaraghavan et al., 2017). However, although studies have detected Rb inactivation by hyperphosphorylation in breast cancer cells resistant to endocrine therapy and in patients’ tumors receiving adjuvant endocrine treatment (Thangavel et al., 2011; Malorni et al., 2016), large clinical trials couldn’t indicate a statistically relevant link between Rb and resistance to CDK4/6 inhibitors (Turner et al., 2019; Finn et al., 2020). An alternative mechanism of resistance to CDK4/6 inhibitors is CDK6 overexpression. *CDK6* amplification mediated resistance to abemaciclib in breast cancer cells (Yang et al., 2017), while elevated CDK6 protein levels were associated with acquired resistance to endocrine treatment (Correction, 2016).

### Combination treatment strategies

Instead of monotherapy, the development of novel therapeutic combinations based on the mechanisms of resistance has been an emerging area. Several preclinical studies and clinical trials proposed the synergistic effect of different agents either with only the CDK4/6 inhibitors or in combination with endocrine therapy. The main two combinatorial strategies involve growth factors that either activate upstream of the cyclin D-CDK4/6-Rb pathway or pathway members, such as mTOR, PI3K, AKT, RAF and MEK, and enhance the cytostatic effect of CDK4/6 inhibitors or the apoptosis of cancer cells (Cheng et al., 1998; Vora et al., 2014; Goel et al., 2016; Herrera-Abreu et al., 2016; Jansen et al., 2017; Formisano et al., 2019; Alves et al., 2021; Lelliott et al., 2021; Zhao et al., 2021). Combination of CDK4/6 inhibitors and immunotherapy is another strategy to induce anti-cancer immune responses by using anti-PD-1/PD-L1 inhibitors (Deng et al., 2018; Schaer et al., 2018; Yuan et al., 2021). A synergistic effect of abemaciclib and the PD-L1 checkpoint blocker led to complete tumor rejection compared to tumor growth delay by the abemaciclib monotherapy and enhanced adaptive and innate immune activation (Schaer et al., 2018).

Induction of autophagy by CDK4/6 inhibitors is a common resistance mechanism in cancer treatment (Chittaranjan et al., 2014; Wang et al., 2016). Therefore, the combinational effect of hydroxychloroquine (HCQ), a late-stage autophagy inhibitor, with palbociclib and abemaciclib is being examined in breast cancer cells, mice, and phase I/II or phase II clinical trials (NCT03774472, NCT04523857, and NCT04841148) (Vijayaraghavan et al., 2017). Co-treatment of palbociclib with HCQ induced irreversible growth inhibition and elevated levels of senescence in breast cancer cells and decreased tumor volume in both the treatment and recovery phase after the treatment was stopped (Vijayaraghavan et al., 2017).

The interplay between CDK4/6 and mitogen-activated protein kinase (MAPK) inhibitors was explored by several studies and clinical trials. Most of the solid tumors with resistance to CDK4/6 inhibitors, are characterized by alterations in MAPK key genes, such as *RAS*, *RAF*, *MEK* and *ERK*, which represent promising therapeutic targets (Stern, 2018). In addition, MAPKs are major regulators of cyclin D1, and abnormal MAPK expression levels lead to cancer progression and resistance to therapies (Plotnikov et al., 2011). This dependence on MAPK activation suggests that combinatorial treatment with MAPK and CDK4/6 inhibitors is a potentially effective cancer treatment strategy. Indeed, preclinical studies revealed that xenograft models with BRAF, KRAS or NRAS mutations were more sensitive to this combinatorial treatment and induced sustained tumor regression (Yadav et al., 2014; Yoshida et al., 2016; Pek et al., 2017; Chen et al., 2018; Kim et al., 2018; Martin et al., 2018; Teh et al., 2018). In addition, numerous clinical trials are performed with this combinatorial treatment in various cancer types by using ribociclib, palbociclib, abemaciclib or dalpiciclib as CDK4/6 inhibitors and different MAPK inhibitors (Table 1).

**TABLE 1:** Clinical trials with CDK4/6 inhibitors in combination with MAPK inhibitors.

CDK4/6 inhibitor	Tumor	Intervation/Treatment	Target	Phase	Clinical trial
Ribociclib (LEE011)	Metastatic Melanoma	Trametinib	MEK	Phase II	NCT02645149
		Standard therapy or clinical trial			
		Supportive care			
Ribociclib (LEE011)	Recurrent Brain Tumors	Trametinib	MEK	Phase I	NCT03434262
		Gemcitabine	chemotherapy agent		
		Sonidegib	Hedgehog signaling pathway inhibitor		
		Filgrastim	treat neutropenia		
Ribociclib (LEE011)	Non-Small Cell Lung Cancer Melanoma	Naporafenib (LXH254)	B- and CRAF	Phase I	NCT02974725
		Rineterkib (LTT462)	ERK1/2		
		Trametinib	MEK		
Ribociclib (LEE011)	EGFR-mutant Non-small Cell Lung Cancer	Trametinib	MEK	Phase I	NCT03333343
		LXH254	RAF		
		Nazartinib (EGF816)	EGFR		
		Capmatinib (INC280)	MET		
		Gefitinib	EGFR		
Ribociclib (LEE011)	Melanoma	Naporafenib (LXH254)	B- and CRAF	Phase II	NCT04417621
		Rineterkib (LTT462)	ERK1/2		
		Trametinib	MEK		
Ribociclib (LEE011)	Solid Tumors Harboring a BRAF V600 Mutation	Encorafenib (LGX818)	BRAF	Phase I/II	NCT01543698
		Binimetinib (MEK162)	MEK		
Ribociclib (LEE011)	Melanoma	Encorafenib (LGX818)	BRAF	Phase II	NCT02159066
		Binimetinib (MEK162)	MEK		
		Infigratinib (BGJ398)	FGFR		
		Buparlisib (BKM120)	pan-class I PI3K		
		Capmatinib (INC280)	MET		
Palbociclib (PD-0332991)	Solid Tumors	Trametinib	MEK	Phase I	NCT02065063
Palbociclib (PD-0332991)	KRAS Mutant Non-Small Cell Lung Cancer	Mirdametinib (PD-0325901)	MEK	Phase I/II	NCT02022982
	Solid Tumors				
Palbociclib (PD-0332991)	Lung Cancer	Binimetinib (MEK162)	MEK	Phase I/II	NCT03170206
Palbociclib (PD-0332991)	Triple Negative Breast Cancer	Binimetinib (MEK162)	MEK	Phase I/II	NCT04494958
Palbociclib (PD-0332991)	Metastatic Colorectal Carcinoma	Binimetinib (MEK162)	MEK	Phase II	NCT03981614
		Trifluridine and Tipiracil Hydrochloride	treat colon, rectal, or stomach cancer		
Palbociclib (PD-0332991)	Advanced Pancreatic and Other Solid Tumors	Ulixertinib (BVD-523)	ERK1/2	Phase I	NCT03454035
Palbociclib (PD-0332991)	Non-Small Cell Lung Cancer	Selumetinib	MEK1/2	Phase II	NCT02664935
	Carcinoma, Squamous Cell	Vistusertib	mTOR		
	Adenocarcinoma	Crizotinib	receptor tyrosine kinases (RTK)		
		AZD4547	FGFR		
		Docetaxel	chemotherapy agent		
		AZD5363	AKT		
		Osimertinib	EGFR		
		Durvalumab	immunotherapy agent		
		Sitravatinib	receptor tyrosine kinases (RTK)		
		AZD6738	ATM/ATR		
Palbociclib (PD-0332991)	Leukemia	Sorafenib	RAF	Phase I	NCT03132454
		Decitabine	chemotherapy agent		
		Dexamethasone	glucocorticoid		
Abemaciclib (LY2835219)	Recurrent Glioblastoma Patients	Temuterkib (LY3214996)	ERK1/2	Early Phase I	NCT04391595
Abemaciclib (LY2835219)	Tumors with BRAF V600E, MEK1, MEK2, ERK and RAF1 mutations	Temuterkib (LY3214996)	ERK1/2	Phase II	NCT04534283
Abemaciclib (LY2835219)	Metastatic Melanoma	Temuterkib (LY3214996)	ERK1/2	Phase I	NCT02857270
	Metastatic Non-small Cell Lung Cancer	Encorafenib (LGX818)	BRAF		
	Colorectal Cancer	Midazolam	benzodiazepine		
	Advanced Cancer	Nab-paclitaxel	antiproliferative agent		
		Gemcitabine	chemotherapy agent		
		Cetuximab	EGFR		
Dalpiciclib (SHR6390)	Luminal Advanced Breast Cancer	SHR7390	MEK1/2	Phase II	NCT04355858
		Famitinib	receptor tyrosine kinases (RTK)		
		SHR3162	PARP		
		Pyrotinib	EGFR/HER2		
		Capecitabine	chemotherapy agent		
		Camrelizumab (SHR-1210)	anti-PD1		
		Everolimus	mTOR		
		Nab paclitaxel	antiproliferative agent		
		SHR2554	EZH2		
		SHR3680	androgen-receptor (AR) antagonist		
		SHR1701	anti-PD-L1/TGF-βRII		
		Selective estrogen receptor degrader or downregulator (SERD)	anti-hormone therapy		
		AI	aromatase inhibitor		
		VEGFi	VEGF		

Even though the majority of patients respond to the standard first-line drug therapy with CDK4/6 inhibitors combined with endocrine therapy, there is an increasing need to validate novel biomarkers to escape intrinsic or acquired multiple resistance mechanisms. Currently developed combinatorial therapies have made remarkable progress in the efficacy of CDK4/6 inhibitors for HR+/HER2- metastatic breast cancer therapy. Further comprehensive research into potential resistance mechanisms may lead to novel combinatorial treatment strategies and consequently the use of CDK4/6 inhibitors for additional diseases.

## Kinase-independent functions

The world of kinases is complicated by the fact that many of them perform kinase-independent functions in addition to their kinase-dependent ones. To stay with the example of RAF kinases: Several kinase-independent functions have been described for CRAF, such as its interaction with the Rho effector Rok-α that affects keratinocyte and fibroblast migration (Ehrenreiter et al., 2005), or its binding to ASK1 that inhibits ASK1’s pro-apoptotic function (Chen et al., 2001). Moreover, the suppression of ERBB3-AKT-driven lung cancer spreading requires both kinase-dependent and independent functions of ARAF. Whereas ARAF suppresses activation of AKT kinase in a kinase-dependent manner, it inhibits the expression of ERBB3 in a kinase-independent manner (Mooz et al., 2022). Here, ARAF-dependent regulation of ERBB3 expression was mediated by the transcription factor KLF5. Not only were we surprised by the different regulatory mechanisms of ARAF with respect to the ERBB3-AKT signaling axis, but also by the observation that ARAF, previously classified more as an oncogene, acts as a tumor suppressor in a subset of lung cancers. We could show that ARAF has a suppressive effect on lung colonization of cells injected into the tail vein of mice and we observed that low expression of ARAF correlates with poor survival of lung cancer patients, highlighting the clinical relevance. Besides ARAF, CRAF has also been shown to have tumor suppressive properties in hepatocellular carcinoma (Jeric et al., 2016). Therefore, to develop rational therapies, it is important to also consider the tissue-specific role of “oncogenic” kinases in a kinase-dependent and kinase-independent manner. Better understanding of how the switch between oncogenic and tumor-suppressive kinase function works will in turn enable a variety of treatment options.

## Pseudokinases

The study of pseudokinases has revealed the ways in which kinase-independent functions can affect biological processes. Approximately 10% of the members forming the human kinase group are explicitly classified as pseudokinases because they lack the necessary catalytic groups (Manning et al., 2002), while others contain multiple somatic mutations (Forbes et al., 2010; Hu et al., 2021) of known or unknown consequences directly or indirectly disrupting the activity of functional motifs. Manning and co-workers (Scheeff et al., 2009) have characterized the integrity and activity of functional motifs in pseudokinases. Particularly, they have predicted the integrity of glycine-rich loops (“intact,” “degraded” or “plausible degradation”) or their variations in atypical kinases. They have also characterized the VAIK, HRD and DFG motifs and predicted whether they are inactive. Pseudokinases were also subclassified into four groups based on their nucleotide-binding properties (Murphy et al., 2014): 1) devoid of detectable nucleotide or cation binding, 2) cation-independent nucleotide binding, 3) cation binding, and 4) nucleotide binding enhanced by cations.

**FIGURE 2:**
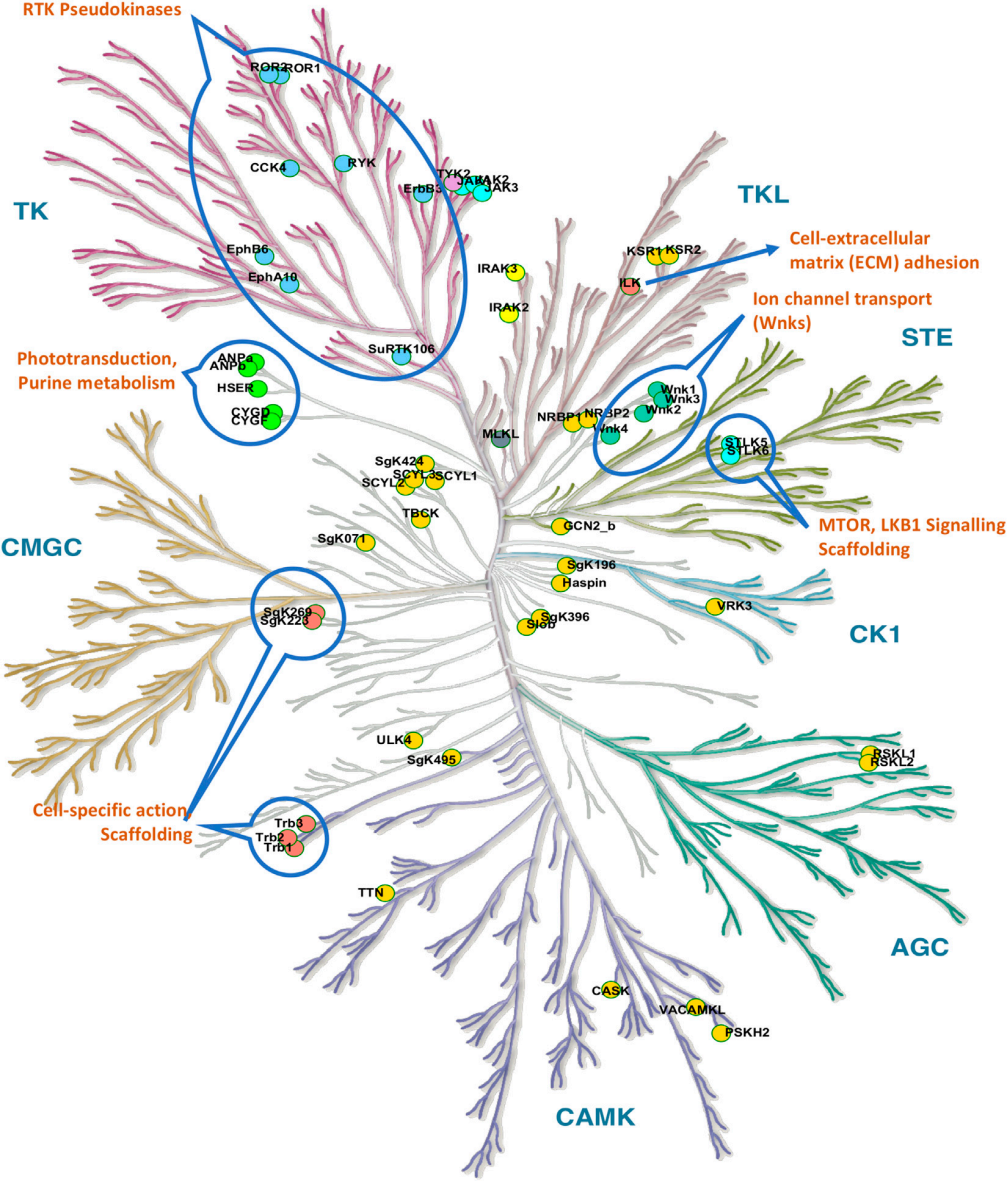
Visualization of pseudokinases. The kinome tree of pseudokinases shows a range of functions including scaffolding (The Tribbles TRIB1-3), cell-specific actions, visual signal transduction (phototransduction), purine metabolism, Ephrin receptors, ion channel transport (Wnk1-4), and others. The pseudokinases ADCK3 (atypical) and PAN3 (other) are not shown. The kinome tree illustration is reproduced from Kinmap portal by Cell Signaling Technology, Inc.

Although pseudokinases lack kinase activity, a growing number of them have been shown to play key roles in regulating various cellular processes (Figure 2), including MAPK signaling, actin polymerization, and the development of malignancies (Fukuda et al., 2009; Scheeff et al., 2009; Ghatak et al., 2013; Toms et al., 2013; Murphy et al., 2015; Stefely et al., 2015; Martin et al., 2022). Three principal mechanisms of kinase-independent actions have emerged, which may also occur in combination in some pseudokinases: 1) Allosteric regulation of kinase activity through heterodimerization of pseudokinase and kinase. 2) Action as scaffolding proteins that recruit signaling proteins to regulate biological endpoints such as cell migration and invasion. 3) Action as molecular switches. Notably, each of these functions can theoretically also occur in kinases in addition to their kinase activity (Jacobsen and Murphy, 2017).

### Allosteric activation by dimerization

The transition from an inactive, monomeric configuration of the kinase domain to an active, dimeric configuration is essential for kinase activation and achieved through an allosteric regulatory mechanism (Engelman et al., 2007; Hu et al., 2013; Lavoie et al., 2014; Dhawan et al., 2016). Pseudokinases are often the result of gene duplication and exhibit substantial homology with their family members, with whom they frequently cooperate (Adrain and Freeman, 2012; Murphy et al., 2017; Ribeiro et al., 2019). Similarly, the ability to allosterically regulate kinases has been preserved in many pseudokinases. For example, the Kinase Suppressors of Ras 1 and 2 (KSR1/2) are well-studied pseudokinases of the RAF family and required for maximal activation of the Raf/MEK/ERK kinase cascade (Rajakulendran et al., 2009; Brennan et al., 2011; Lavoie et al., 2018). They do this by acting both as a scaffold bringing together the components of the cascade (see section “scaffolding function”) and by allosterically activating their RAF kinase family members. Dimerization and subsequent autophosphorylation of RAF kinases is a prerequisite for their kinase activity and controlled by GTP-bound RAS engaged through their RAS-binding domains (RBD). Besides homodimerization, RAF family members may also be activated by dimerizing with KSR1/2. KSR1/2 lack RBDs and thus the heterodimerization relies on a different regulatory principle that rather involves the RAF substrate MEK. Indeed, binding of MEK to the kinase domain of KSR1/2 induces conformational changes in KSR1/2 that drive in turn “side-to-side” heterodimerization with BRAF enabling the allosteric transactivation of BRAF by KSR1/2 (Lavoie et al., 2018). It is assumed that BRAF-KSR dimerization serves to establish the active conformation of BRAF as a prerequisite for subsequent cis-auto- phosphorylation of the BRAF activation loop, which is required for full activation of the dimer (Hu et al., 2013). Of note, the MEK molecule that induces BRAF-KSR dimerization is not the one targeted by the activated BRAF but rather a free “substrate” MEK molecule is engaged for phosphorylation (Lavoie et al., 2018). The FDA-approved MEK inhibitor trametinib, which disrupts RAF-MEK binding, was recently shown to stabilize KSR-MEK binding by directly engaging KSR at the MEK interface (Khan Z. M. et al., 2020). Thus, KSR is a critical factor to be considered in the evaluation and development of clinical MEK inhibitors. KSR1 is itself an advantageous target for the development of new therapeutics. KSR1 can be functionally inhibited with small molecules (e.g., APS-2-79) that stabilize KSR1 in an inactive conformation that blocks heterodimerization with BRAF (Lozano et al., 2003; Dhawan et al., 2016). However, efficacy and potential of this approach for the treatment of Ras-driven cancers remain to be demonstrated.

Another well-studied example of a pseudokinase that allosterically regulates its kinase-active family members is ErbB3/HER3, a well-documented ligand-dependent dimer- ization partner of epidermal growth factor receptor (EGFR), ErbB2/HER2, and c-Met (Engelman et al., 2007). By forming an asymmetric kinase dimer, HER3 stabilizes its dimer partner in the active conformation (Zhang et al., 2006; Jura et al., 2009). ErbB3/HER3 overexpression is associated with lung, colon, gastric, and other cancers (Amin et al., 2010) and has been also found to be responsible for resistance to HER2, IGF1R, and EGFR inhibitors in the treatment of several types of cancers (Erjala et al., 2006; Sergina et al., 2007; Desbois-Mouthon et al., 2009; Miller et al., 2009).

### Molecular switches

A dynamic switch-like conformational change is inherent to the activation of eukaryotic kinases leading to phosphorylation of their substrates. It is typically controlled by phosphorylation of the activation loop phosphate (Zhang et al., 2009) but can also occur in response to the binding of partners. Mixed lineage kinase domain-like (MLKL) is a paradigm example of a pseudokinase that acts as a molecular switch but when turned on results in multimerization and necroptotic cell death rather than phosphorylation-dependent signaling (Zhang et al., 2009; Murphy et al., 2013; Petrie et al., 2018). Necroptosis is a lytic form of programmed cell death characterized by the rupture of cells, often triggering inflammatory responses. It is associated with numerous human diseases, including cancer and inflammatory diseases, but has no function in development, making it an attractive therapeutic target. Key molecules of necroptosis are the kinases RIPK1 and RIPK3 that form the necroptotic complex (also called necrosome) (Cho et al., 2009; Li et al., 2012), and their phosphorylation target MLKL, which is considered the executioner of necroptosis (Zhang et al., 2009; Wu et al., 2013; Su et al., 2014). MLKL comprises an N-terminal four-helix bundle (4HB) domain connected to a C-terminal pseudokinase domain (PsKD), which lacks two of the three conserved catalytic residues (Zhang et al., 2009; Su et al., 2014). In the inactive state MKLK is monomeric and the 4HB domain, which is required for lipid engagement and membrane permeabilization, engages in inhibitory intramolecular interactions with the PsKD. Upon stimulation, RIPK3-mediated phosphorylation of the PsKD (Thr356 and Ser357) leads to the switch-like conformational change that frees the 4HB domain from intramolecular inhibition and results in the formation of pro-necroptotic MLKL tetramers that translocate to the cell membrane to cause cell expansion, rupture of the membrane and cell death (Zhang et al., 2009; Cai et al., 2014; Chen et al., 2014; Su et al., 2014; Wang et al., 2014; Petrie et al., 2018). While in some diseases (ischemic injury, inflammation, etc.) the goal is to inhibit necroptosis, in cancer therapy the goal is to activate it. In contrast to apoptosis, necroptosis triggers inflammation or causes an innate immune response through the release of damage-associated molecular patterns (DAMPs) and might thus trigger antitumor immunity in cancer therapy to defend against tumor progression (Su et al., 2016; Gong et al., 2019). Though lacking any catalytic activity, MLKL has retained its ability to bind nucleotides and it has been found that ATP-binding to MLKL as well as MLKL PsKD mutants, including some identified in cancers (Forbes et al., 2017), stabilized the monomeric OFF state. In contrast, mutations mimicking RIP3-mediated phosphorylation within the PsKD promoted the ON state (Petrie et al., 2018), however, in human MLKL they were not sufficient to induce necroptosis in a cell model. This knowledge has been the basis for the development of small molecules (Rübbelke et al., 2020; Rübbelke et al., 2021).

Another example for a switch-like function is provided by the Janus kinase (JAK) family of non-receptor tyrosine kinases that transduce extracellular cytokine signals through the JAK-STAT pathway and controls biological processes such as immunity, cell division, and cell death particularly in hematopoiesis. Besides playing critical roles in host defense and autoimmunity, JAKs are associated with cancers especially hematologic malignancies (James et al., 2005; Levine et al., 2005; Erdogan et al., 2022). JAKs contain both an active kinase domain (Jak homology 1, JH1) and a pseudokinase domain (Jak homology 2, JH2) in tandem. Structural studies and molecular dynamics simulations on JAK2 revealed that JH2 inhibits the kinase activity of JH1 by intramolecular interactions that stabilize the kinase domain in an inactive state (Saharinen et al., 2000; Saharinen and Silvennoinen, 2002; Shan et al., 2014). The switch to the active state occurs upon cytokine binding leading to a receptor rearrangement that facilitates JAK2 *trans*-phosphorylation of activation-loop tyrosines 1007–1008 in JH1. The importance of this switch is illustrated by the fact that mutations in JH2 are the most common activating somatic mutations underlying hematologic malignancies (Haan et al., 2010). Structural studies revealed that the most frequent of these mutations (V657F in Jak1 and V617F in Jak2) promotes the rearrangement of the pseudokinase domain allowing JH2 to adopt the active conformation (Toms et al., 2013). In fact, only recently Glassman *et al.* resolved the structure of full-length active JAK1 bound to intracellular domain regions of a cytokine receptor and investigated the structural impact of the oncogenic mutation V657F, showing that this mutation is positioned at the central pseudokinase interdimer interface (Glassman et al., 2022).

### Scaffolding function

Pseudokinases are often multidomain proteins able to recruit multiple components of a signaling pathway to efficiently elicit a specific cellular response. For example, besides its function as allosteric activator of RAF kinases, KSR also acts as a scaffold providing docking sites for MEK, ERK, 14-3-3 proteins, caveolin-1, IMP, and phosphatases that collectively regulate flux through the RAF-MEK-ERK cascade. Notably, consistent with the function as scaffold excess levels of KSR disrupt signaling as members of the cascade are “diluted” rather than concentrated on a single KSR molecule.

Another intriguing example of a pseudokinase scaffold is the Tribbles family (TRIB1, TRIB2 and TRIB3) (Eyers et al., 2017), which is implicated in a wide variety of cancers as well as drug resistance. The structural studies on the family member TRIB1 revealed that the C-terminal tail binds to a pocket formed by helix αC in the N-lobe of the pseudokinase domain in a way that resembles an autoinhibitory conformation of conventional kinases precluding “substrate” binding by the highly conserved DQXVP motif (Murphy et al., 2015; Jamieson et al., 2018). One of the best characterized proteins that binds to this motif is the E3 Ub-ligase COP1, but it is also reported that the tribble family act as cell-type specific scaffolds for MEK/MAPK, AKT and NFκB signaling using other motifs (Wei et al., 2012; Yu et al., 2019). Unlike for COP1, the underlying mechanisms here are less well understood but may also involve autoinhibitory interactions between C-terminal tail and the pseudokinase domain.

The oncogenic PEAK family (PEAK1/SgK269, PEAK2/SgK223/Pragmin, and PEAK3) also functions as scaffolds for different cellular processes, particularly morphology, and migration (O'Rourke and Daly, 2018; Roche et al., 2019). Their molecular and structural features enable PEAKs to operate in a spatially and temporally regulated way: Besides being multidomain proteins offering a variety of docking sites and regulatory phosphorylation sites, PEAK proteins dimerize in a unique way *via* their split helical dimerisation (SHED) domains (Patel et al., 2017; Ha and Boggon, 2018) making the pseudokinase domain accessible from the outside to allow additional homo- and heterotypic interactions that are key to their function(s). The molecular mechanisms by which PEAKs regulate cytoskeletal and adhesion signaling are complex and context-dependent rendering therapeutic targeting challenging.

Finally, the integrin-linked kinase (ILK) is an evolutionarily conserved, intracellular pseudokinase scaffold with widespread expression and a central component of cell-extracellular matrix (ECM) adhesions where it facilitates bidirectional signaling between the ECM and intracellular sites (Hannigan et al., 2005; Fukuda et al., 2009; Qin and Wu, 2012). To this end, ILK forms a heterotrimer (the PINCH-ILK-parvin complex) through protein-protein interactions *via* multiple sites including the pseudoactive site. ILK activity is stimulated by adhesion to the ECM as well as growth factors in a PI3K-dependent manner. ILK overexpression induces epithelial-mesenchymal transition (EMT) by inhibiting E-cadherin expression and generates a tumorigenic phenotype through activation of nuclear β-catenin. It also promotes cell survival by stimulating the phosphorylation of AKT on Ser473. ILK activation promotes VEGF expression in tumor cells and has a crucial role in endothelial activation and angiogenesis. Therefore, ILK is one of the most versatile pseudokinases that can drive cell proliferation, anchorage and growth-factor independence, angiogenesis, cell death evasion (which may trigger MLKL-driven necroptosis), cell or tissue invasion and metastasis.

Inhibition of ILK has been a key strategy for many cancer treatments (Martin et al., 2022). Due to the complexity of protein-protein interactions and a plethora of affected pathways, these programs exhibit various levels of success in their development—these include chronic lymphocytic leukemia (CLL), breast cancer, and various cancer cell lines *in vitro*. Knockout of ILK sensitizes breast cancer to SRC inhibitors such as bosutinib (Beetham et al., 2022). When used independently, SRC inhibitor programs have not been successful but a combination therapy regime involving simultaneous inhibition of ILK and SRC would be more promising in future.

### Challenges in targeting pseudokinases therapeutically

Pseudokinases were long considered undruggable. However, as indicated above, solid evidence suggests that pseudokinases are very similar to canonical kinases in their “active/ON” conformation, which offers approaches for inhibitor development. The most promising pseudokinases to target were those that retained their ability to bind nucleotides, as the ATP-binding site is considered the most “druggable” pocket in protein kinases. Pseudokinases functioning by allosteric activation of their dimer partners are currently the predominant target for interventions using small-molecules that act as ATP-competitive inhibitors binding to their pseudoactive site (Dhawan et al., 2016; Kim, 2020; Lu et al., 2020; Mace and Murphy, 2021). Yet, also the scaffolding and switch-like functions of pseudokinases could be addressed in this way, examples being TRIB2 (Bailey et al., 2015; Machado et al., 2020) and MLKL (Hildebrand et al., 2014), respectively.

The major pharmacological challenge of targeting the pseudoactive sites with small molecules is basically the same as for active kinases and, that is, selectivity and effectiveness (Ma et al., 2016). Moreover, many pseudokinases did not retain their nucleotide-binding ability, such as the PEAK family, or even lack a defined binding pocket that could be targeted by ATP-competitive molecules. Therefore, other binding surfaces outside the kinase-domain are searched for (Rübbelke et al., 2021), and novel therapeutic strategies, such as ProTACs or hydrophobic tagging (HyT) that induce degradation of specific proteins are also being pursued (Kung and Jura, 2019).

The analysis of pseudokinases for biomarker validation and drug development is greatly aided by the use of databases that allow the review of the pathophysiological effects of somatic mutations. For example, the ProKinO ontology resource (Gosal et al., 2011; McSkimming et al., 2015) provides a downloadable list of annotated pseudokinase domains and sequences. Somatic mutations of pseudokinases in cancer are available from the KinaseMD and COSMIC databases (Forbes et al., 2010; Forbes et al., 2017; Hu et al., 2021). Despite the tremendous recent developments in multiomics analysis, there is still a dark kinome that deserves further attention which shall open further avenues of therapeutic interventions. Further characterization of the biology of these understudied kinases and pseudokinases are much needed to cater the unmet medical needs in cancers. It is equally important to elucidate the role of these druggable family members in the immune system, as targeting these kinases should not dampen the immune responses for effective antitumor therapy.
